# The National COVID Cohort Collaborative: Clinical Characterization and Early Severity Prediction

**DOI:** 10.1101/2021.01.12.21249511

**Published:** 2021-01-23

**Authors:** Tellen D. Bennett, Richard A. Moffitt, Janos G. Hajagos, Benjamin Amor, Adit Anand, Mark M. Bissell, Katie Rebecca Bradwell, Carolyn Bremer, James Brian Byrd, Alina Denham, Peter E. DeWitt, Davera Gabriel, Brian T. Garibaldi, Andrew T. Girvin, Justin Guinney, Elaine L. Hill, Stephanie S. Hong, Hunter Jimenez, Ramakanth Kavuluru, Kristin Kostka, Harold P. Lehmann, Eli Levitt, Sandeep K. Mallipattu, Amin Manna, Julie A. McMurry, Michele Morris, John Muschelli, Andrew J. Neumann, Matvey B. Palchuk, Emily R. Pfaff, Zhenglong Qian, Nabeel Qureshi, Seth Russell, Heidi Spratt, Anita Walden, Andrew E. Williams, Jacob T. Wooldridge, Yun Jae Yoo, Xiaohan Tanner Zhang, Richard L. Zhu, Christopher P. Austin, Joel H. Saltz, Ken R. Gersing, Melissa A. Haendel, Christopher G. Chute, N3C Consortium

**Affiliations:** 1. Section of Informatics and Data Science, Department of Pediatrics, University of Colorado School of Medicine, University of Colorado, Aurora, CO, USA; 2. Department of Biomedical Informatics, Stony Brook University, Stony Brook, NY, USA; 3. Stony Brook University, Stony Brook, NY, USA; 4. Palantir Technologies, Denver, CO, USA; 5. The University of Michigan at Ann Arbor, Ann Arbor, MI, USA; 6. University of Rochester Medical Center, Rochester, NY, USA; 7. Johns Hopkins University School of Medicine, Baltimore, MD, USA; 8. Sage Bionetworks, Seattle, WA, USA; 9. University of Kentucky, Lexington, KY, USA; 10. Real World Solutions, IQVIA, Cambridge, MA, USA; 11. Observational Health Data Sciences and Informatics, New York, NY, USA; 12. University of Alabama at Birmingham, Birmingham, AL, USA; 13. Translational and Integrative Sciences Center, Oregon State University, Corvallis, OR, USA; 14. Department of Biomedical Informatics, University of Pittsburgh, Pittsburgh, PA, USA; 15. TriNetX, Cambridge, MA, USA; 16. North Carolina Translational and Clinical Sciences Institute (NC TraCS), University of North Carolina at Chapel Hill, Chapel Hill, NC, USA; 17. Stony Brook University, Stony Brook, NY, USA; 18. University of Texas Medical Branch, Galveston, TX, USA; 19. Oregon Clinical and Translational Research Institute, Oregon Health & Science University, Portland, OR, USA; 20. Tufts Medical Center Clinical and Translational Science Institute, Tufts Medical Center, Boston, MA, USA; 21. National Center for Advancing Translational Sciences, National Institutes of Health, Bethesda, MD, USA; 22. Translational and Integrative Sciences Center, Dept. of Molecular Toxicology, Oregon State University, Corvallis, OR, USA; 23. Schools of Medicine, Public Health, and Nursing, Johns Hopkins University, Baltimore, MD, USA

## Abstract

**Background:**

The majority of U.S. reports of COVID-19 clinical characteristics, disease course, and treatments are from single health systems or focused on one domain. Here we report the creation of the National COVID Cohort Collaborative (N3C), a centralized, harmonized, high-granularity electronic health record repository that is the largest, most representative U.S. cohort of COVID-19 cases and controls to date. This multi-center dataset supports robust evidence-based development of predictive and diagnostic tools and informs critical care and policy.

**Methods and Findings:**

In a retrospective cohort study of 1,926,526 patients from 34 medical centers nationwide, we stratified patients using a World Health Organization COVID-19 severity scale and demographics; we then evaluated differences between groups over time using multivariable logistic regression. We established vital signs and laboratory values among COVID-19 patients with different severities, providing the foundation for predictive analytics. The cohort included 174,568 adults with severe acute respiratory syndrome associated with SARS-CoV-2 (PCR >99% or antigen <1%) as well as 1,133,848 adult patients that served as lab-negative controls. Among 32,472 hospitalized patients, mortality was 11.6% overall and decreased from 16.4% in March/April 2020 to 8.6% in September/October 2020 (p = 0.002 monthly trend). In a multivariable logistic regression model, age, male sex, liver disease, dementia, African-American and Asian race, and obesity were independently associated with higher clinical severity. To demonstrate the utility of the N3C cohort for analytics, we used machine learning (ML) to predict clinical severity and risk factors over time. Using 64 inputs available on the first hospital day, we predicted a severe clinical course (death, discharge to hospice, invasive ventilation, or extracorporeal membrane oxygenation) using random forest and XGBoost models (AUROC 0.86 and 0.87 respectively) that were stable over time. The most powerful predictors in these models are patient age and widely available vital sign and laboratory values. The established expected trajectories for many vital signs and laboratory values among patients with different clinical severities validates observations from smaller studies, and provides comprehensive insight into COVID-19 characterization in U.S. patients.

**Conclusions:**

This is the first description of an ongoing longitudinal observational study of patients seen in diverse clinical settings and geographical regions and is the largest COVID-19 cohort in the United States. Such data are the foundation for ML models that can be the basis for generalizable clinical decision support tools. The N3C Data Enclave is unique in providing transparent, reproducible, easily shared, versioned, and fully auditable data and analytic provenance for national-scale patient-level EHR data. The N3C is built for intensive ML analyses by academic, industry, and citizen scientists internationally. Many observational correlations can inform trial designs and care guidelines for this new disease.

## Introduction

As of mid-December 2020, severe acute respiratory syndrome associated with coronavirus-2 (SARS-CoV-2) has infected more than 70 million people and caused more than 1.6 million deaths worldwide^[a]^. SARS-CoV-2 can cause coronavirus disease of 2019 (COVID-19), a condition characterized by pneumonia, hyperinflammation, hypoxemic respiratory failure, a prothrombotic state, cardiac dysfunction, substantial mortality, and persistent morbidity in some survivors. Few FDA-authorized therapeutics are available, and vaccine deployment has been slow. Progress in COVID-19 research has been slowed by lack of broad access to clinical data. Investigators in the United Kingdom^1^ and Denmark^1,2^ have performed person-level analytics across their populace, but the U.S. has not had this capacity. A large, multi-center, representative clinical dataset has been desperately needed by U.S. clinicians, scientists, health systems, and policy-makers to develop predictive and diagnostic computational tools and to inform critical decisions.

To address these gaps, N3C was formed to accelerate understanding of SARS-CoV-2 and demonstrate a novel approach for collaborative data sharing and analytics during a pandemic. The National COVID Cohort Collaborative (N3C)^3^ is comprised of members from the NIH Clinical and Translational Science Awards (CTSA) Program and its Center for Data to Health (CD2H), the IDeA Centers for Translational Research^[b]^, the National Patient-Centered Clinical Research Network (PCORNet, pcornet.org), the Observational Health Data Sciences and Informatics (OHDSI, ohdsi.org) network, TriNetX (trinetx.com), and the Accrual to Clinical Trials (ACT, actnetwork.us/National) network.

Here we provide a detailed clinical description of the largest cohort of U.S. COVID-19 cases and representative controls to date. This cohort is racially and ethnically diverse and geographically distributed. We demonstrate its impact by 1) evaluating COVID-19 severity and risk factors over time and 2) using machine learning (ML) to develop a clinically useful model that accurately predicts severity using data from the first day of hospital admission.

## Methods

### Cohort Definition and Outcome Stratification

Because of the broad inclusion criteria, N3C includes cases and appropriate controls for varied analyses including both outpatients and inpatients (Supplemental Table 1). N3C includes patients with any encounter after 1/1/2020 with 1) one of a set of *a priori*-defined SARS-CoV-2 laboratory tests or 2) a “strong positive” diagnostic code or 3) two “weak positive” diagnostic codes during the same encounter or on the same date prior to 5/1/2020. The cohort definition is publicly available on GitHub.^[c]^ For N3C patients, encounters in the same health system beginning on or after 1/1/2018 are also included to provide information about pre-existing health conditions (“lookback data”). See Supplemental Methods for information about N3C architecture, data ingestion, and integration.

**Figure 1:**
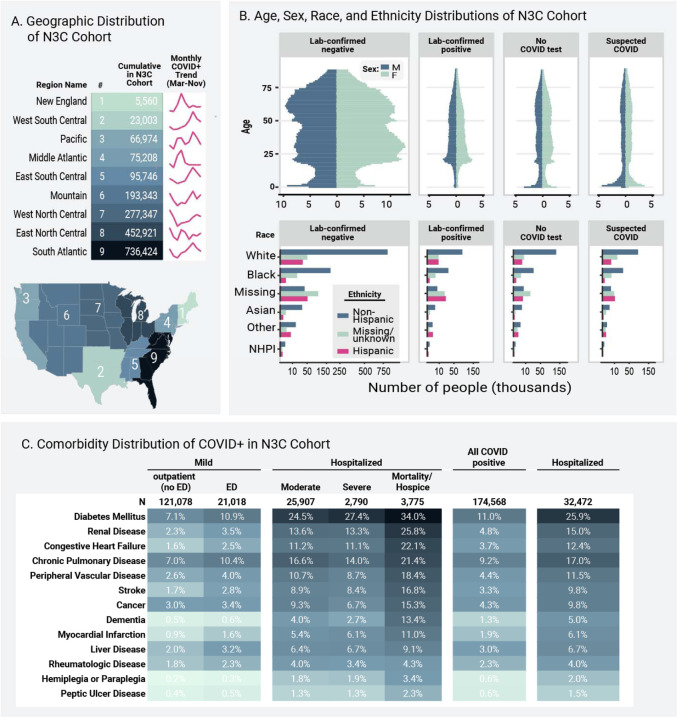
Geographic, Age, Sex, Race, Ethnicity, and Comorbidity Distributions of N3C Cohort. **Figure 1a** shows the representation of each U.S. subregion in the overall (N = 1,926,526) cohort. Trend lines show the accumulation of each subregion’s sample size of lab confirmed positive cases over 2020. The Southeast, Mid-Atlantic, and Midwestern regions are the most heavily represented, but all regions have substantial patient counts. **Figure 1b** shows the age, sex, race, and ethnicity distributions of the overall N3C cohort, stratified by the N3C phenotype groups (publicly available on GitHub^[c]^). Racial and ethnic minorities are well-represented. COVID = coronavirus disease. NHPI = Native Hawaiian or Pacific Islander. **Figure 1c** shows comorbidity distributions for the laboratory-confirmed positive adult cohort (N = 174,568). See Supplemental Methods for comorbidity definitions. We stratified patients using the Clinical Progression Scale (CPS) established by the World Health Organization (WHO) for COVID-19 clinical research, see Table 1^4^. Severity assigned by patient-specific encounter maximum severity. No ED = outpatient only without emergency department visit, ED = emergency department visit, moderate = hospitalized without invasive ventilation or extracorporeal membrane oxygenation (ECMO), severe = hospitalized with invasive ventilation or ECMO, mortality/hospice = hospital mortality or discharge to hospice.

We conducted a retrospective cohort study of adults ≥ 18 years old at the 34 N3C sites whose data 1) have completed harmonization and integration (see Supplemental Methods), 2) were released for analysis, and 3) included the necessary death and mechanical ventilation information (Supplemental Figure 1). In order to demonstrate the scope of N3C, Figure 1a–b and Supplemental Table 1 are based on the entire cohort. All subsequent analyses include only patients with a positive SARS-CoV-2 laboratory test (polymerase chain reaction [PCR] or antigen) (Table 1).

### Hospital Index Encounter and Clinical Severity

We defined a single index encounter for each laboratory-confirmed positive patient using a pre-specified algorithm (Supplemental Methods). We stratified patients using the Clinical Progression Scale (CPS) established by the World Health Organization (WHO) for COVID-19 clinical research.^4^ We placed patients into strata defined by the maximum clinical severity during their index encounter (Table 1). We collapsed some WHO CPS categories due to data limitations (e.g. some sites do not submit fraction of inspired oxygen [FiO_2_]).

### Variable Definition and Statistical Methods

We defined or identified existing concept sets in the Observational Medical Outcomes Partnership (OMOP) common data model (CDM) for each clinical concept (e.g. laboratory measure, vital sign, or medication, see Supplemental Methods). We validated each concept set with input from informatics and clinical subject matter experts. All concept sets and analytic pipelines are fully reproducible and will be made publicly available. We tested time trends using linear regression and differences between groups using multivariable logistic regression. See Supplemental Methods for additional information including software packages used.

### Machine Learning Methods

We developed models to predict patient-specific maximum clinical severity: hospitalization with death, discharge to hospice, invasive mechanical ventilation, or extracorporeal membrane oxygenation (ECMO) versus hospitalization without any of those. To avoid immortal time bias, we only included patients with at least one hospital overnight. We split the hospitalized laboratory-confirmed positive cohort into randomly selected 70% training and 30% testing cohorts stratified by outcome proportions and held out the testing set. We chose a broad set of potential predictors present for at least 15% of the training set (Supplemental Table 2). The input variables are the most abnormal value on the first calendar day of the hospital encounter. When patients did not have a laboratory test value on the first calendar day, we imputed normal values for specialized labs (e.g. ferritin, procalcitonin) and the median cohort value for common labs (e.g. sodium, albumin) (Supplemental Table 2). We compared several analytical approaches with varying flexibility and interpretability: logistic regression +/− L1 and L2 penalty, random forest, support vector machines, and XGBoost (github.com/dmlc/xgboost).

We internally validated models and limited overfitting using 5-fold cross-validation and evaluated models using the testing set and area under the receiver operator characteristic (AUROC) as the primary metric. Secondary metrics included precision/positive predictive value, recall/sensitivity, specificity, and F1-measure. Because SARS-CoV-2 outcomes have improved over time^5^, we evaluated model performance overall and for March-May 2020 and June-October 2020. See Supplemental Methods.

### Role of the funding source

The primary study sponsors are multiple institutes of the U.S. National Institutes of Health. The National Center for Advancing Translational Sciences is the primary steward of the N3C data, and created the underlying architecture of the N3C Data Enclave, manages the Data Transfer Agreements and Data Use Agreements, houses the Data Access Committee, and supports contracts to vendors (see conflicts of interests section) to help build various aspects of the N3C Data Enclave. Employees of the NIH and of the contracting companies are included as authors of the manuscript and participated in the writing and decision to submit the manuscript. Please see the author contribution section for details.

## Results

### Study Cohort

As of December 7, 2020, data from 34 sites was harmonized and integrated into the N3C release set. The cohort includes data about 1,926,526 patients (Supplemental Table 1). The cohort derives from all U.S. geographic regions, but is more concentrated in the Southeast, Mid-Atlantic, and Midwest (Figure 1a). The age, sex, race, ethnicity, and insurance payer distributions (Figure 1b and Supplemental Table 1) indicate a diverse patient cohort that is representative of many segments of the U.S. population. Importantly, African-American and Hispanic patients, who have suffered disproportionately from COVID-19^6^, are represented in sufficient numbers to support robust subgroup analyses, pathophysiologic hypothesis generation, and testing of algorithms and models to avoid bias (Table 1).

Supplemental Tables 3a and 3b show the cohort stratified by CDM and strengths and weaknesses of each CDM. Figure 1a shows cohort geographic distribution evolution during 2020.

Of the overall cohort, 174,568 adults (9.1%) had a positive SARS-CoV-2 PCR or antigen test at a site with death and ventilation data available (Table 1). Antigen tests represent <5% of a single site’s positive tests. All other positive patients had positive PCR tests.

### Clinical Course and Mortality

**Figure 2.**
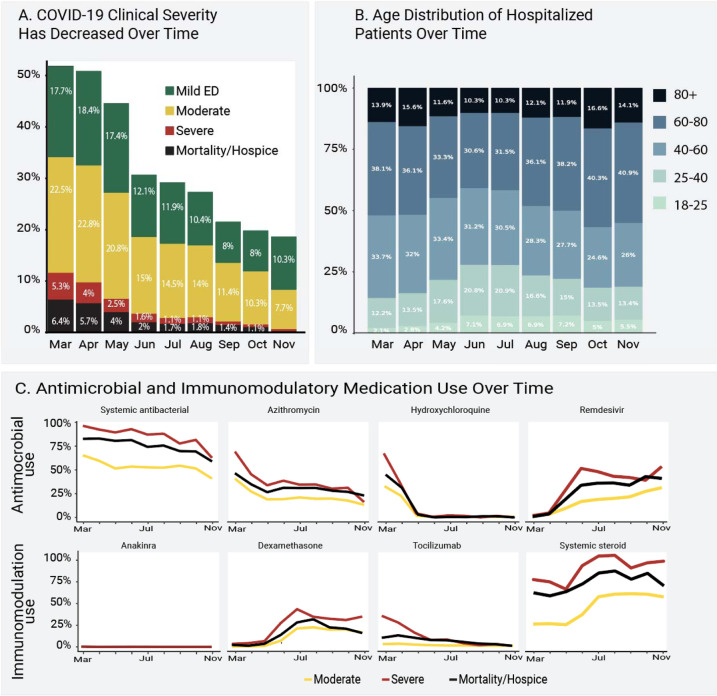
Clinical Severity, Age, and Antimicrobial and Immunomodulatory Medication Use Over Time **Figure 2a** shows the distribution of patient-specific encounter maximum severity among hospitalized patients during 2020. Mortality and invasive ventilation or extracorporeal membrane oxygenation (“Severe”) have decreased steadily, monthly trend p = 0.002. Strata assigned using the Clinical Progression Scale (CPS) established by the World Health Organization (WHO) for COVID-19 clinical research (hospital mortality or discharge to hospice [black], invasive ventilation or extracorporeal membrane oxygenation [red], hospitalized without any of those [yellow], or emergency department visit only [green], see Table 1^4^). The percentage of patients from each month is shown over each severity group bar. **Figure 2b** shows how the age distribution of hospitalized patients has changed during 2020. The percentage of patients from each month is shown over each age bracket bar. Older patients (darker blue) were more prominent in the spring and the fall, with more younger patients (lighter blue/teal) in the summer. **Figure 2c** shows the evolution of antimicrobial and immunomodulatory treatment regimens for hospitalized patients (top 3 severity strata, see Table 1) during 2020.

Of those with a positive test, 32,472 (18.6%) were hospitalized. The median length of hospital stay was 5 days (IQR 2 to 10). Mortality (including discharge to hospice) was 11.6% among hospitalized patients (Table 1). Others have reported that inpatient mortality has decreased over time^7^. We confirm this: inpatient mortality decreased from 16.4% in March and April to 8.6% in September and October (P for monthly linear trend 0.002). Our data also show that clinical severity has shifted toward less invasive mechanical ventilation and/or ECMO as the pandemic has progressed (Figure 2a).

### Demographics, Comorbidities, and Obesity

The age distribution for hospitalized patients was older during spring 2020, younger during the summer, and older again in the fall (Figure 2b). Lookback data that allowed calculation of comorbidities was present for 49% of hospitalized patients. Of hospitalized patients, 41% had at least one comorbid condition; the most common was diabetes mellitus (25.9%, Figure 1c). Mean body mass index (BMI) was 30 or above for all severity groups (Table 1). In a multivariable logistic regression model, age, male sex, liver disease, dementia, African-American and Asian race, and obesity (BMI > 30) were independently associated with higher patient-specific maximum clinical severity (invasive ventilation, ECMO, death, or discharge to hospice versus none of those, Supplemental Table 4). Interestingly, rheumatologic disease and blood type AB were protective. This analysis was conducted only to provide inference about previously reported risk factors and occurred after the prediction model was built, see below.

### Vital Sign and Laboratory Measurements

**Figure 3.**
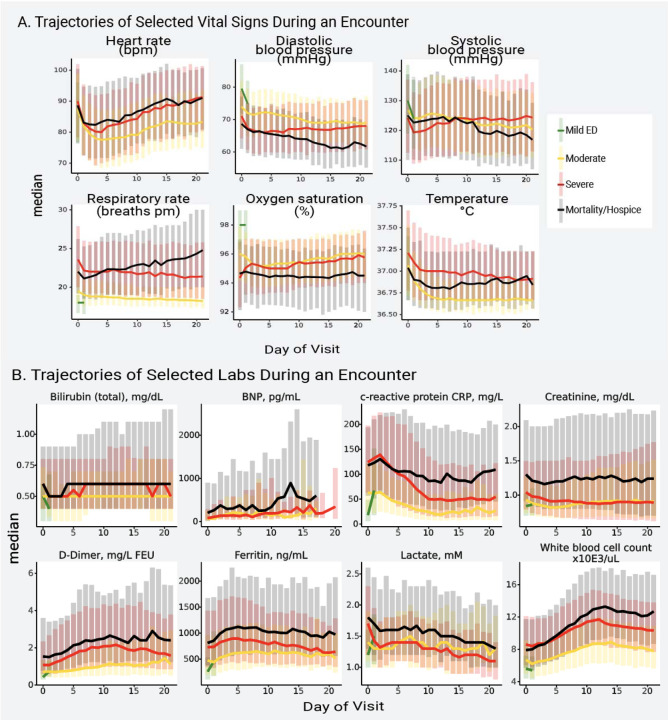
Trajectories of Vital Signs and Laboratory Tests During a Hospital Encounter Figure 3a shows the median (line) and interquartile range (bars) of each vital sign on each hospital day, stratified by patient maximum severity (hospital mortality or discharge to hospice [black], invasive ventilation or extracorporeal membrane oxygenation [red], hospitalized without any of those [yellow], or emergency department visit only [green], see Table 1). Figure 3b shows the median (line) and interquartile range (bars) of each laboratory test on each hospital day, stratified by the same severity groups. BNP = brain natriuretic peptide.

As a hospital encounter progressed, those who ultimately developed higher clinical severity (invasive ventilation, ECMO, or death) tended to have progressively more abnormal (higher) mean heart rate (HR), respiratory rate (RR), and temperature than those who did not (Figure 3a). Mean diastolic blood pressure (DBP) and oxygen saturation (SpO_2_) among those who ultimately died continued to become more abnormal (lower) while those who were invasively ventilated or on ECMO became more normal (higher, Figure 3a). Early in the hospital encounter, mean values of DBP, SpO_2_, and widely used measures of inflammation (C-reactive protein [CRP] and ferritin), immunologic activation (white blood cell count, WBC), fibrinolysis (D-dimer), oxygen delivery (lactate), and renal function (creatinine) were more abnormal among those who ultimately required invasive ventilation or ECMO than those who did not (Figures 3a and 3b). These findings support the hypothesis that clinical severity can be predicted using information available early in a hospital course (see prediction models).

Other measurements (e.g. sodium, platelet count, lymphocyte count) show potential utility as early outcome predictors, as their values near the beginning of a hospital encounter tend to separate patients with lower and higher maximum clinical severity (Supplemental Figure 2). Mean values of brain natriuretic peptide were low early in hospital encounters but showed meaningful spikes between hospital days 10 and 15. This is consistent with reports of the timing of cardiac failure in COVID-19^8^. Overall, patients with more abnormal nadir and/or peak values of several vital signs and laboratory measurements were more often represented in higher severity groups (invasively ventilated, ECMO, or death; Supplemental Figures 3a–b). CRP, ferritin, D-dimer, WBC, and IL-6 have been identified by the WHO as key biochemical parameters for a core COVID-19 outcome set^4^. These were measured in 44–94% of hospitalized patients, except IL-6 (7.6%). A relatively small number of hospitalized patients had blood type data (9.1%, Supplemental Figure 4).

### Treatments

Usage of antimicrobial and immunomodulatory medications has changed dramatically over time (Figure 2c). Overall, 66.2% of the hospitalized cohort received at least one antimicrobial, with significant treatment regimen heterogeneity (Supplemental Figure 5a and Supplemental Table 5a). Patients who received invasive ventilation and ECMO received more antimicrobials overall (Supplemental Figure 5a). Antivirals with potential activity against SARS-CoV-2 were given to 16.7% (remdesivir) and 0.6% (lopinavir/ritonavir) of hospitalized patients. At least one immunomodulatory medication was given to 41.5% of hospitalized patients, also with wide variation in treatment regimen (Supplemental Figure 5b and Supplemental Table 5a). More patients received hydrocortisone, methylprednisolone, and prednisone than dexamethasone (Supplemental Table 5a). The trial indicating survival benefit from dexamethasone was published in July 2020.^9^ Other steroids also have modestly supportive clinical trial data.^10^

Of the hospitalized cohort, 14.0% received any invasive respiratory support (mechanical ventilation or inhaled or systemic pulmonary vasodilators, Supplemental Table 5b). Similarly, 8.3% received medications for cardiovascular support or ECMO and 3.2% received dialysis or continuous renal replacement therapy.

### Severity Prediction

**Figure 4.**
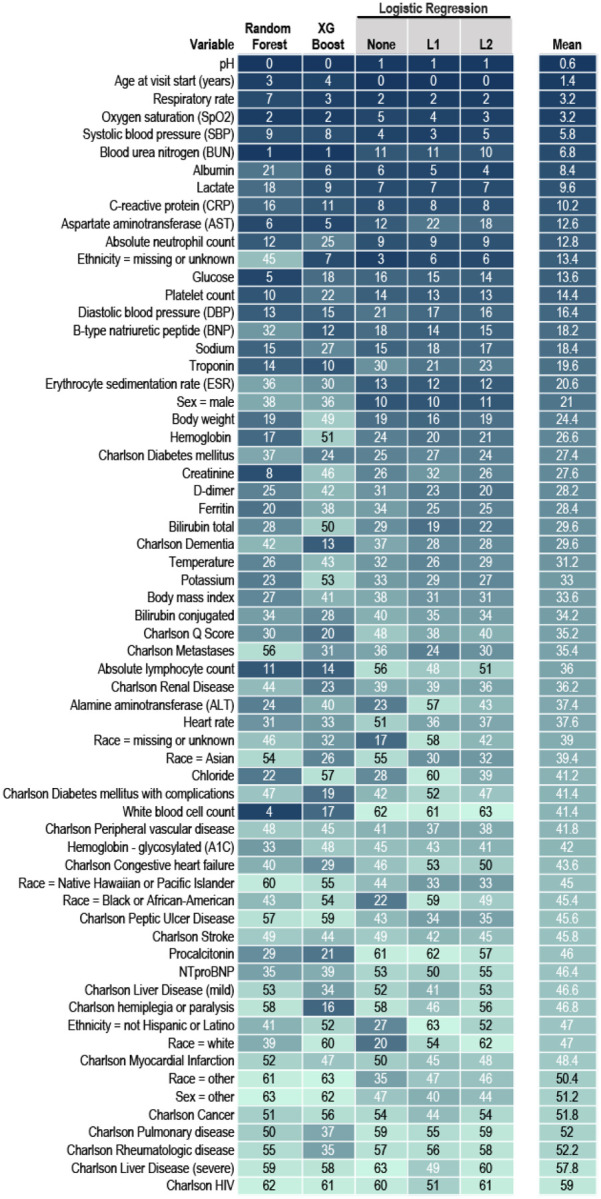
Variable Importance in the Machine Learning Models Predicting Clinical Severity The 64 machine learning (ML) model input variables are listed by their mean variable importance rank across ML model types. Each column is a ML model type. Logistic regression is shown without penalization and with L1 and L2 penalties. The table cells show a heat map with darkest (blue) representing highest variable importance and lightest (teal) representing lower variable importance. See Methods and Supplemental Methods for details about variable definitions, model construction, and testing. NTproBNP = N-Terminal-prohormone B-type Natriuretic Peptide.

We developed several models that accurately predict a severe clinical course using data from the first hospital calendar day (Supplemental Figure 6 and Supplemental Table 6). The models with the best discrimination of severe versus non-severe clinical course were built using XGBoost (AUROC 0.87) and random forest (AUROC 0.86). Both are flexible nonlinear tree-based models that provide interpretability with a variable importance metric (Figure 4). Importantly, discrimination by the two models was stable over time (March-May 2020 and June-October 2020, Supplemental Table 6). This indicates that the models did not train on health care processes only typical during the pandemic’s chaotic first wave. Commonly collected variables (age, SpO_2_, RR, blood urea nitrogen, systolic blood pressure, and aspartate aminotransferase) were among the inputs with the highest variable importance for both models (Figure 4).

## Discussion

This manuscript characterizes the largest U.S. COVID-19 cohort to date. We have confirmed a month-over-month decrease in COVID-19 inpatient mortality and invasive ventilation rates since March 2020. We developed accurate ML models to predict clinical severity based only on information available on the first calendar day of admission. The most powerful predictors in these models are patient age and widely available vital sign and laboratory values. These models can be the basis for generalizable clinical decision support tools. We also established expected trajectories for many vital signs and laboratory values among patients with different clinical severities. Expected trajectories can contribute to clinician decision-making about what a patient will need.

Site heterogeneity in the distribution of predictors of severe COVID-19 disease including age, race, ethnicity, and existing comorbidities (e.g. diabetes) has complicated interpretation of their independent impact on outcomes. Like others, we found that age, male sex^1^, African-American race^6,11^ and obesity^12,13^ were associated with greater clinical severity. Associations of liver disease and dementia with COVID-19 severity have also been reported^14,15^. We found that patients with rheumatologic disease had lower clinical severity. This is consistent with reports that after adjustment for age, diabetes, and renal impairment, patients with rheumatologic disease on some treatment regimens have lower risk of hospitalization^16^. Increased risk of intubation and death has been inconsistently found among patients with blood types AB, A, and B relative to type O.^17–19^ In contrast, we found that blood type AB was protective.

We also found significant treatment regimen heterogeneity for inpatients with COVID-19. Some medications have fallen out of favor (e.g. hydroxychloroquine, azithromycin); others are the subject of ongoing studies (e.g. anakinra, tocilizumab). For most treatments, the balance of risks and benefits has not been evaluated rigorously in randomized controlled trials. Ongoing monitoring for adverse effects in observational data like N3C will be important.

The N3C has unique features that distinguish it from other COVID-19 data resources. First, it harmonizes data from a very large number of clinical sites (73 have signed data transfer agreements to date). This is important because significant site-level variation in critical metrics such as invasive ventilation and mortality has been reported.^20–23^ Central curation ensures that N3C data are robust and quality-assured across sites. This is in contrast to the known challenges of relying on site-level CDM quality assurance processes in distributed networks (e.g. OHDSI, PCORnet). Most U.S. reports of COVID-19 clinical characteristics, disease course, treatments, and outcomes come from a single hospital or health system^6,22^ in a single geographic region. Another network has reported a large COVID-19 cohort, but the patient-level data is not centralized and thus is less amenable to machine learning^24^.

Developed under the intense time pressure of a health crisis, earlier data aggregation efforts^1,21,25–28^ may not have been designed to support future research. The N3C Data Enclave^3^ provides transparent, easily shared, versioned, and fully auditable data and analytic provenance. This is a key advantage, as a lack of auditable data and analytic provenance has resulted in retraction of high-profile COVID-19 publications.^29,30^

N3C users should bear in mind its limitations. Because the data are aggregated from many health systems and 4 CDMs that vary in granularity, some sites have systematic missingness of some variables (see Supplemental Methods). Detailed respiratory support information such as oxygen flow, FiO_2_, and ventilator settings (typically recorded in EHR flowsheets) is not fully available. Orders related to limitations in care such as “do not attempt resuscitation” (DNAR) are not yet present in N3C. Some inpatient mortality in our study likely occurred in patients who had DNAR orders in place. Exclusion of those patients might improve severity model prediction. Finally, exact time of laboratory values is inconsistently provided by sites, so labs are standardized to calendar day, but not time of day.

In conclusion, N3C is a nationally representative, transparent, reproducible, harmonized data resource that enables effective and efficient collaborative observational COVID-19 research. N3C is built for intensive machine learning analyses by academic, industry, and citizen scientists internationally. We have demonstrated its utility by developing a clinically useful patient severity predictor.

## Supplementary Material

Supplement 1

## Figures and Tables

**Table 1:** SARS-CoV-2 Laboratory-Confirmed Positive Cohort Characteristics and Clinical Course

	Mild Outpatient WHO Severity 1–3	Mild_ED Outpatient with ED visit WHO Severity ~3	Moderate Hospitalized without invasive ventilation WHO Severity 4–6	Severe Hospitalized with invasive ventilation or ECMO WHO Severity 7–9	Hospital Mortality or Discharge to Hospice WHO Severity 10
**N**	121,078	21,018	25,907	2,790	3,775
**Age (mean +/− SD)**	41.1 (17.2) n=121078	43.4 (16.8) n=21018	55.0 (19.1) n=25907	57.0 (15.4) n=2790	71.8 (14.7) n=3775
**Sex**					
** Female**	65,435	11,410	13,396	1,089	1,564
** Male**	55,526	9,605	12,506	1,697	2,211
** Other** *	117	20 or fewer	20 or fewer	20 or fewer	0
**Race**					
** White or Caucasian**	70,330	7,786	10,739	1,020	1,912
** Black or African-American**	14,616	6,351	8,003	869	1,101
** Native Hawaiian or Pacific Islander**	267	40	66	20 or fewer	20 or fewer
** Asian**	2,778	564	717	86	120
** Other**	1030	403	373	51	48
** Missing/Unknown**	32,057	5,874	6,009	757	584
**Ethnicity**					
** Hispanic**	18,539	5,312	5,145	610	476
** Non-Hispanic**	80,188	12,510	17,313	1,789	2,779
** Missing/Unknown**	22,351	3,196	3,449	391	520
**Insurance Payer**					
** Medicare**	2,480	906	2,852	308	823
** Commercial**	11,718	2,277	1,984	227	237
** Medicaid**	2,945	1,590	1,974	242	294
** Other**	115,480	18,576	22,876	2,409	3,124
**Body Size**					
** Body Mass Index (mean +/− SD)**	30.1 (7.6) n=39836	31.2 (7.8) n=9552	31.0 (9.0) n=16489	32.9 (9.4) n=1862	29.5 (8.7) n=2440
** Weight, kg (mean +/− SD)**	86.3 (23.7) n=47284	87.3 (23.7) n=13511	88.6 (26.0) n=20068	95.5 (26.8) n=2349	84.6 (26.7) n=3106
**Clinical course**					
** Hospital LOS, median (IQR)**			6.6 (8.9) n=2590	27.5 (26.1) n=2790	14.0 (23.3) n=3775

SARS-CoV-2 = severe acute respiratory syndrome associated with coronavirus-2. ED = Emergency Department. WHO = World Health Organization. ECMO = extracorporeal membrane oxygenation. LOS = length of stay. We stratified patients using the Clinical Progression Scale (CPS) established by the World Health Organization (WHO) for COVID-19 clinical research.^4^ Severity assigned by patient-specific encounter maximum severity.

* Other includes non-binary, no matching concept, and no information. Per N3C policy, we censored any cells with 1–20 patients and replaced them with “20 or fewer.”

## Footnotes

[a].
https://coronavirus.jhu.edu/map

[b].
https://www.nigms.nih.gov/Research/DRCB/IDeA/Pages/IDeA-CTR.aspx

[c].
https://github.com/National-COVID-Cohort-Collaborative/Phenotype_Data_Acquisition
